# The Largest Drought in American History: Funding for Science Is Drying Up

**DOI:** 10.1371/journal.pntd.0002351

**Published:** 2013-08-29

**Authors:** A. Desiree LaBeaud, Hannah McKeating

**Affiliations:** Children's Hospital Oakland Research Institute, Oakland, California, United States of America

Funding for neglected tropical diseases (NTDs) and other areas of science, while steady, has failed to keep up with the rate of inflation over the last several years, scaring scientists (and future scientists) enough to abandon research careers altogether. In 2011, the Obama administration granted $32.1 billion for the National Institutes of Health (NIH) (up $1 billion; 3.2% increase) and $7.4 billion for the National Science Foundation (NSF) (up $550 million; 8% increase) [1]. This budget increase has since been eliminated, and inflation continues to slowly erode the purchasing power of remaining NIH dollars [2].

This year's fiscal cliff has made funding difficult to acquire for all, but science and its lobbyists predict disastrous results. The current sequestration is estimated to result in 5–9% cuts at the NIH, resulting in about 2,500 fewer grants for the NIH and another 1,500 fewer grants for the NSF in 2013 [3], [4]. Effects will also be felt in the Department of Defense research enterprise. Stable scientific funding has become an unattainable goal in this period of budget uncertainty.

Funding has declined in all areas of science, including NTD research, due to stagnant federal budgets. Infectious diseases continue to get less support than other disease groups, such as cancer. The U.S. invested $4.7 billion into research and development for the National Institute of Allergy and Infectious Disease (NIAID) in 2010 [5], compared to the National Cancer Institutes' (NCI) $5.1 billion [6]. Among infectious diseases, the NTDs secure significantly less funding than the “big three”, i.e., malaria, tuberculosis, and HIV. Although the fall-off in funding may reflect fewer dollars available, fewer grants submitted, and/or fewer grants succeeding in these areas, NTD funding constitutes only 10.2% of grant funding for infectious diseases, compared 71% for the “big three” [7]; however, even “big three” support is suffering, falling off 72–77% between 2007 and 2009 [7], [8]. The decline in funding cannot completely be attributed to the budget problem.

For new scientists, securing an independent project R01 grant has become a nearly impossible task in the current funding climate. Every funded grant must be in the top 10% of applications, an incredibly difficult achievement in the first few years of a research career [9]. In 2011, only 14% of funding went to young researchers. In the recent past between 1998 and 2003, Congress doubled the NIH budget, and even gave more financial support to help new scientists [9], [10]. Programs such as the NIH Loan Repayment Program (LRP), which was initiated in 1988, encourage promising researchers and scientists to pursue research careers by repaying up to $35,000 of their qualified student loan debt each year [11]. The NIH has also taken steps to improve the funding outlook for young scientists through the “Early Independence Award Program” [12] and “Pathway to Independence Awards” (K99/R00) [13]; however, only a few percentile points are given to boost new investigator prospects for major (R01) funding. It seems that much more could be done to prevent the exodus of young, talented scientists.

The funding drought affects not only young scientists, but also other scientists at all levels of training. Mid-career investigators who were initially able to secure their first R01s are now finding it impossible to renew their grants. Often, after building successful laboratories with trained personnel, they are forced to let their human capital and laboratory investments go because of inability to secure ongoing funding. Not only does this make for an uncertain and unstable career, it can also stifle transformative scientific research. Small, safe steps become the research norm instead of dramatic leaps forward in knowledge [14].

Placing research in particular peril is the growth of an anti-science movement in America. The danger of this movement is that it distracts the research agenda and leaves the public ignorant of very real threats to American public health. As a result, America is now much less of a competitor in science and technology, although it still leads today. There is however, a major risk of falling behind China, India, Japan, and the majority of Europe in terms of advancement and development in science, technology, and education [15], [16].

NIH funding is a long process that begins with planning committee meetings and the President's fiscal year budget plan, which then settles into the House annual appropriations bill. The House and Senate draft funding legislation for the NIH and other federal agencies through 13 appropriation subcommittees [17]. Once funds are allocated and approved, the NIH implements its budget by funding grants and contracts. Although Congress decides the fate of the NIH's budget, only 2% of elected representatives have a background in science [18]. In 2008, after many invitations, presidential candidates Barack Obama and John McCain refused to even acknowledge the call to the Science Debate 2008, a set of over 3,400 questions to be answered [18], [19]. With 85% of the American people wanting presidential candidates to discuss scientific topics during debates [18], why are our elected officials and presidential candidates avoiding the topic? Although the public turns to the media for factual information, scientific journalism has been reduced significantly. The *Washington Post* eliminated its scientific journalism department in 2008, like many other newspaper companies [18].

Our dramatic refusal to talk about scientific issues within our government or engage scientists in public forums is mirrored in our youth. In colleges and universities, science and technology produce the fewest graduates each year. Numbers of graduating college students have increased by 50%, but the number of students graduating with science and technology degrees remains flat [20]. Why would undergraduates pursue a career in science or technology when the career outlook is so bleak? Business has become the most popular degree (in 2012, there were 358,000 majors) sought after, while science and research (18,300 majors) lags far behind [21]. Funding has been cut, finding a stable position is now increasingly difficult, and, if a position is secured, such jobs pay poorly. Why would young brilliant minds pursue a career in science or technology? A scientific career has become a scary, forbidding option, with only 4% of America's workforce dedicated to science and research. Because American public schools are failing to emphasize math and science in K–12 programs, many science and technology facilities are looking abroad for their new employees instead of hiring American graduates. Nearly 45% of PhD graduates under the age of 45 are foreign born, and these represent a significant proportion of those getting hired [15].

America once was the world's leader in science, research, and development, but because of our repeated mistakes we face losing our reputation in scientific standing. Some propose solutions to improve the situation by: 1) matching global standards for math and science in U.S. K–12 education; 2) encouraging more U.S. residents to pursue careers in math, science, and engineering; 3) encouraging more scientists to pursue politics; 4) improving the quality and quantity of scientific reporting to the public; and 5) recognizing America's strength in science and technology and protecting our legacy with appropriate federal budgets [13]. Highlighting scientific issues and discoveries in the media can reach the public and inspire the next generation to choose careers in science and research. Meanwhile, for NIH funding of new investigators, a threshold percentage of 20% of new R01s could be allotted to new investigators, with a return to the policy that allows two cycles of application revisions, and a larger percentile boost for new investigator scores. Scientists need to engage the public in their research and lobby their congresspersons (or become congresspersons) for greater research funding via NIH, NSF, and Department of Defense research programs. As scientists, we need to muster the confidence to advocate for our own futures and the future of science in America.
